# Toxic Effects of Excess Vitamins A, B6, and Folic Acid on the Nervous System

**DOI:** 10.1155/bn/7888243

**Published:** 2025-08-06

**Authors:** Yu Sun, Xiaofeng Yu, Yiliang Teng, Yanping Sun

**Affiliations:** ^1^Department of Neurology, The Affiliated Hospital of Qingdao University, Qingdao, China; ^2^Department of Neurology, Qingdao Eighth People's Hospital, Qingdao, China; ^3^Department of Neurology, Qingdao Shinan Integrated Chinese & Western Medicine Hospital, Qingdao, China

**Keywords:** folic acid, nervous system, pyridoxine, retinoic acid, toxicity, vitamins

## Abstract

As one of the seven primary nutrients in the human body, vitamins are vital to maintaining good health. In recent years, there has been a completely new understanding of vitamins, and researchers have conducted more thorough investigations on them. These compounds, once viewed simply as supplemental nutrients, are now believed to play a more complex and critical role in human health. On the other hand, ingesting too much vitamins may cause negative health effects. Multiple studies have demonstrated a strong correlation between neurological problems and excessive vitamin intake. The purpose of this paper is to review the toxic effects of excessive vitamin intake on the nervous system, focusing on vitamin A and some of the B vitamins. It also analyzes the relationship between excessive vitamin intake and neurological dysfunction by reviewing the research findings in recent years, mainly including their possible mechanisms of action, clinical manifestations, and preventive measures, to provide ideas and inspiration for the subsequent clinical research.

## 1. Introduction

In the late 19th century, it was believed that a physiologically complete diet required only adequate protein, carbohydrates, fats, inorganic salts, and water. This view was gradually overturned between 1880 and 1912, when Cornelis Adrianus Pekelharing observed that animals fed purified proteins, carbohydrates, fats, inorganic salts, and water failed to grow healthily or even lost weight and died unless their diets were supplemented with small amounts of milk. He deduced that milk contained unknown substances that, even in minute quantities, were still essential to normal growth and health. Frederick Gowland Hopkins agreed with these opinions. In 1911, Casimir Funk, a Polish biochemist, isolated a biologically active substance in rice bran that cured pigeons with polyneuritis. He named the substance “vitamine” because Funk incorrectly believed it was an amine that was necessary for life. Although it was later proven that the substance was not an amine, the name was modified to “vitamin.” In the decades that followed, the science surrounding vitamins continued to make significant developments, and all 13 vitamins had been discovered by 1941 [1–3]. They are categorized by their nature into fat-soluble and water-soluble vitamins. Fat-soluble vitamins include four types, A, D, E, and K. There are nine water-soluble vitamins, including vitamin C and eight B vitamins [4].

Today, almost half of Americans take vitamins and dietary supplements, and as many as 80% believe that vitamins and supplements are safe [5]. With their widely known efficacy in avoiding disease and maintaining nutritional health, there is an increasing need for vitamin supplements [6]. However, while pursuing the benefits of vitamins, some people may neglect the possible negative effects of excessive or inappropriate usage of vitamins that may be harmful to their health, which must be considered seriously [7]. In this paper, we will review the relevant literature and select vitamin A from fat-soluble vitamins and some B vitamins from water-soluble vitamins to investigate the effects of vitamin overdose on the nervous system, to provide a theoretical basis for the study of vitamin overdose and neurological adverse events, as well as some ideas and references for the future study of clinical treatment methods.

## 2. Vitamin A

### 2.1. Concept and Intake of Vitamin A

Vitamin A, the first fat-soluble vitamin discovered in history, plays an important role in several physiological processes, including visual function, epithelial tissue growth and differentiation, bone growth, immune system regulation, reproductive health, and fetal development [8]. There are two forms of vitamin A: retinoids and pro–vitamin A carotenoids. Retinoids are the original form of vitamin A and are mainly found in animal foods, such as animal liver, cod liver oil, cream, whole milk, poultry, and eggs, whereas pro–vitamin A carotenoids are mainly found in plant foods, especially orange and red vegetables, and fruits, such as carrots, sweet potatoes with red hearts, chili peppers, mangoes, and persimmons [9]. Carotenoids, mainly including beta-carotene, alpha-carotene, and beta-cryptoxanthin, can be converted to vitamin A in the body, and beta-carotene is one of the most prevalent nutritional supplements among adults in the United States [6].

The tolerable upper intake level (UL) for vitamin A in adults is 3000 *μ*g RE/day, and the recommended dietary allowance (RDA) for adults is 800 *μ*g RE/day [10]. Retinol equivalents (REs), which translate to 1 *μ*g of retinol or 3.33 international units (IUs) of vitamin A activity as retinol, are the unit of measurement for vitamin A activity [11]. As a fat-soluble vitamin, vitamin A is eliminated more slowly and accumulates more readily in the body than water-soluble vitamins. It is stored as retinyl palmitate as the major storage form in the liver and adipose tissue [9]. Therefore, fat-soluble vitamins are more likely than water-soluble vitamins to accumulate to harmful levels in the body when taken in excess, which may result in toxicity related to the nervous system. An authoritative measure for determining vitamin A nutritional status is the total hepatic vitamin A concentration, which is the sum of the free and esterified retinol and is expressed in terms of liver tissue weight. The steady retinol isotope dilution (RID) approach, which is at the forefront of indirect assays for evaluating the total vitamin A status of the human body, is used to indirectly determine the hepatic retinol concentration. The cutoff point for excessive hepatic retinol concentrations is still up for debate. Based on data from the RID study, the BOND expert panel proposed a provisional grading threshold with clinical guidance in 2016. Retinol concentrations have a threshold of > 1 *μ*mol/g when hepatic vitamin A stores are high, and it increases to > 10 *μ*mol/g when vitamin A toxicity is attained [10].

In order to prevent the risk of stacked toxicity, the safe upper limit of vitamin A intake during retinoid treatment must be closely monitored. Depending on the specific circumstances (e.g., dosage, course of therapy, and other medications), the doctor may recommend additional restriction or monitoring. The total amount of vitamin A intake (including diet and supplements) should not exceed the UL.

### 2.2. Clinical Manifestations Associated With Vitamin A Overdose

Excessive intake of vitamin A may result in acute or chronic toxic consequences. Acute vitamin A toxicity symptoms may include headache, nausea, vomiting, sleepiness, altered mental status, elevated intracranial pressure, and hypercalcemia; chronic patients may also exhibit teratogenicity, liver damage, and bone abnormalities [9, 12]. If the poisoning remains undetected, it may eventually cause coma and death owing to increased intracranial pressure [13].

#### 2.2.1. Teratogenicity

13-*cis*-Retinoic acid (i.e., isotretinoin) is a classic vitamin A derivative widely used in the treatment of acne and one of the most well-known teratogens [14]. As an important signaling molecule, retinoic acid (RA) is essential for organismal development, and the overactivation of the RA signaling pathway may have toxic effects on neurodevelopment. Exogenous RA has been found in studies using zebrafish as a model to significantly impact the neurodevelopment of zebrafish embryos, resulting in lower hatching rates, morphological abnormalities, disrupted expression of stem cell and neuronal marker genes, and increased apoptosis [15].

In humans, exposure to retinoids has been linked to abnormal embryonic development. A study of retinoid use in pregnant women from 2004 to 2019 showed that the medicine was associated with higher rates of malformations and adverse pregnancy outcomes. Given its potential teratogenicity, contraception is generally recommended during treatment and for 3 years after discontinuation [16]. Further studies have explored the effects of different forms of RA on cell differentiation using a human neural stem cell model, revealing that high levels of ATRA and 9-*cis*-retinoic acid inhibit neural network formation, providing a scientific basis for assessing the potential threat of retinoids in the environment to human health [17]. It has also been suggested that systemic use of isotretinoin has not found evidence of its direct contribution to major birth defects, but attention still needs to be paid to preventing inappropriate use of the drug during pregnancy [18]. In animal research, topical third-generation retinoids have a relatively low teratogenic risk, but more epidemiologic data are needed to confirm the safety of topical usage in human pregnancy [19]. Researchers have gradually turned their attention in recent years to exploring the potential negative consequences of isotretinoin in men of childbearing age, and there are no conclusions in this area that are supported by convincing evidence [14].

#### 2.2.2. Mental Disorders

In the 30-year-long data collected by the FDA from 1997 to 2017, reports of adverse psychiatric reactions following isotretinoin usage were documented in approximately 18,000 patients, with depression having the largest number of reports and anxiety ranking third [20]. According to the retrospective study, 29.2% of adolescents who used isotretinoin to treat acne had mental health issues, and 16.2% developed new psychiatric symptoms. Patients having a history of mental disorders were more likely to exhibit psychological symptoms such as low mood and mood fluctuations than those without a history of mental disorders [21]. Another study also found that adolescent patients taking isotretinoin for the treatment of vulgaris acne may experience subtle mood changes and other typical mood symptoms [22]. According to all of these studies, patients should have their moods regularly checked while undergoing therapy, with those who have a history of mental illnesses and those with recently developed diseases receiving extra care.

Multiple studies contradict the above conclusions about how isotretinoin treatment for acne affects patients' mental health. Among patients treated for acne in Taiwan, there was no significant difference in the risk of developing psychiatric disorders among those who took isotretinoin compared to those who did not, and then the study concluded that taking isotretinoin does not increase the risk of developing psychiatric disorders [23]. Kridin and Ludwig's study demonstrated that isotretinoin treatment not only reduced the incidence of a variety of mental disorders but also did not significantly increase the chances of suicide attempts in the treated patients compared to the control group. The isotretinoin-treated groups all showed a trend toward a lower risk of mental illness than the control group [24]. However, some academics have argued that Kridin and Ludwig's study has many limitations, and the association between isotretinoin, depression, and suicide should be considered with caution [25]. Even if the conclusions are controversial, there is no doubt that the mental health burden of the disease and the additional mental health impact of the drug's possible physical side effects should be taken into account when evaluating the possible adverse consequences of isotretinoin treatment [26, 27].

Vitamin A therapy triggers depressive behaviors and impairs synaptic plasticity in the hippocampus of rats. The hippocampus is a part of the brain that is crucial for mood regulation, memory, and cognitive functions. Vitamin A acid reduces neuronal excitability in the hippocampus by affecting receptor homeostasis and promoting the synthesis of the inhibitory neurotransmitter gamma-aminobutyric acid (GABA), which impacts linked cognitive and emotional functions [28]. Although the detailed mechanisms of how excess RA affects gene expression and neuroplasticity in the hippocampus are unclear, this work offers a potential avenue for enhancing hippocampal neuroplasticity in order to cure excess RA-induced depression.

A significant proportion of the 123 patients treated with isotretinoin reported excessive sleepiness, but further research is required to confirm the association between isotretinoin and hypersomnia. The study has some limitations, including unknown rates of hypersomnia prior to treatment, excessive sleepiness based on self-report, and insufficient follow-up [29]. Some studies dispute this, stating that isotretinoin has no meaningful effect on sleep apnea and sleep quality 3 months after treatment [30].

There are also some case reports of isotretinoin-induced psychiatric problems. An 18-year-old male suffered a psychotic manic episode after using isotretinoin to treat acne, despite having no personal or family history of psychosis [31]. Another patient exhibited visual hallucinations after taking oral isotretinoin, but the hallucinatory symptoms disappeared when the medicine was discontinued, and no other plausible explanations were detected [32].

#### 2.2.3. Pseudotumor Cerebri

Pseudotumor cerebri syndrome (PTCS), also known as idiopathic intracranial hypertension (IH), is a syndrome that manifests as high intracranial pressure, which causes headache, blurry vision, disorientation, and cognitive impairment. The syndrome also occurs with vitamin A overdose as well as therapy with isotretinoin, and the mechanism may be that vitamin A metabolites hinder the reabsorption of cerebral spinal fluid through gene regulation by ATRA in the meninges and choroid plexus [9, 33].

A 52-year-old lady was hospitalized after using 20 mg of oral isotretinoin every day for 2 months to treat rosacea, according to a case reported by Reifenrath et al. in 2023. This is the first report that tentatively correlates OCT results with IH under systemic isotretinoin monotherapy [34]. The observed temporal correlation between isotretinoin therapy and IH is insufficient to establish a clear causative link. Nonetheless, the investigators believe that the use of isotretinoin still played a crucial role in this case. The case published by Douglas et al. demonstrates that topical application of third-generation retinoids may also contribute to increased intracranial pressure. This is the first report of topical RA resulting in the manifestation of fulminant PTCS [35].

## 3. Vitamin B

The B vitamin family consists of eight members: thiamine (B1), riboflavin (B2), niacin (B3), pantothenic acid (B5), vitamin B6, folic acid (B9), and cobalamin (B12). The B vitamins are classified not by their chemical similarity, but rather based on their water-soluble nature and connected functions as coenzymes in cells. They serve as coenzymes for a variety of enzymatic processes that support various aspects of cellular physiological functions, including the major functions of the brain and nervous system [4]. Some of the B vitamins that are closely associated with the neurological system are chosen to be discussed in this review.

### 3.1. Vitamin B6

#### 3.1.1. Concept and Physiologic Function of Vitamin B6

Vitamin B6 is a water-soluble vitamin found in both plant and animal foods, such as meat, chicken, fish, nuts, beans, and whole grains. In addition, people can increase their intake of vitamin B6 by ingesting various forms of dietary supplements [36]. The recommended daily intake for adults is 1.3–1.9 mg [5]. Vitamin B6 exists naturally in three forms: pyridoxine (PN), pyridoxamine (PM), and pyridoxal (PL), each of which produces its own phosphorylated derivative in the body, namely, pyridoxine 5⁣′-phosphate (PNP), pyridoxamine 5⁣′-phosphate (PMP), and pyridoxal 5⁣′-phosphate (PLP). The most biologically active form, PLP, is an enzyme cofactor connected to multiple enzymatic activities related to the metabolism of carbohydrates, proteins, and lipids as well as the production of neurotransmitters, and is involved in more than 150 biochemical reactions in the body [37]. PLP-dependent enzymes also play a role in the manufacture of neurotransmitters, such as glutamic acid decarboxylase (GAD), which produces the inhibitory neurotransmitter GABA, and serine hydroxymethyltransferase (SHMT), which produces glycine [38].

#### 3.1.2. Vitamin B6 Intake

Up to this point, there is no clear consensus on the toxic exposure threshold for vitamin B6, and current guidelines from various agencies differ greatly. Based mostly on case reports of reversible sensory neuropathy following prolonged doses of more than 1000 mg/day, the UL for vitamin B6 set by the Institute of Medicine of the US National Academy of Sciences is 100 mg/day (which is approximately 75 times the recommended daily intake) [39]. The European Food Safety Authority (EFSA) set a UL for vitamin B6 for adults (including pregnant and lactating women) of 12 mg/day in 2023, based on a systematic evaluation of the association between vitamin B6 and peripheral neuropathy. For infants and children, an isovelocity proportionality method was used based on the UL for adults [40].

There is a phenomenon of significant individual differences in the metabolic processes of vitamin B6 in the human body, which helps to explain the differences in sensitivity to vitamin B6 toxicity exhibited by different people [41]. Paluszny and Qiu described the instance of an adult patient who took a multivitamin containing 6 mg of vitamin B6 per day for 10 years, resulting in 3 years of progressive peripheral neuropathy, which gradually resolved after discontinuing the vitamin supplements on the advice of a physician [42]. This instance demonstrates that even at dosages lower than the normal EFSA guidelines, long-term vitamin B6 consumption can cause neurotoxicity. In fact, using vitamin B6 supplements at levels above the UL tends to trigger toxic effects, and the higher the dose, the higher the risk of developing PN and the shorter the time required [43]. However, the potential risk of toxicity at supplementation doses lower than the recommended dietary intake may involve other factors, such as the duration of supplementation, dietary composition, medication use, and genetic background. In any case, the individualized metabolic profile of vitamin B6 has become an undeniably important topic that needs to be addressed.

Based on the existing research findings, the following vitamin B6 safety recommendations are provided. First, for the generally healthy population, the relatively conservative EFSA recommendation of no more than 12 mg/day is recommended. Second, particular populations, such as pregnant and breastfeeding mothers and people with metabolic disorders, should have specialized nutritional evaluations. In clinical practice, individuals receiving long-term vitamin B6 supplementation therapy are advised to be monitored by a physician. Supplementation should be stopped if neurological symptoms including sensory anomalies appear, and patients should be encouraged to get help right once for a professional assessment. These guidelines seek to strike a compromise between the possible danger of neurotoxicity and the nutritional benefits of vitamin B6 supplements. More long-term, large-sample clinical studies are needed to further validate and confirm the precise thresholds for safe vitamin B6 intake.

#### 3.1.3. Mechanism of Vitamin B6 Toxicity

The precise mechanism underlying vitamin B6 poisoning remains unclear despite the growing number of case reports. There are six forms of vitamin B6. PLP is the biologically active form, whereas PN is the inactive form most commonly found in dietary supplements. But which vitamin form is specifically linked to vitamin B6 toxicity in excess?

In a 2017 study, Vrolijk et al. ventured the hypothesis that when PN-containing supplements are taken at high doses or for prolonged periods, inactive PN accumulates and competitively inhibits enzymes that depend on active PLP, leading to peripheral neuropathy [44]. Two enzymes that require PLP as a cofactor, tyrosine decarboxylase and alanine aminotransferase, were used to investigate the impact of inactive PN on the enzymes. Tyrosine decarboxylase catalyzes the decarboxylation of tyrosine to produce tyramine, while alanine aminotransferase catalyzes the transfer of l-alanine amino group to *α*-ketoglutarate. PN decreased the enzymes' activity by 65% and 40%, respectively. Other vitamin types either stimulate or do not affect enzyme activities. In vitamin B6 deficiency, enzymes dependent on PLP as a cofactor would similarly be partially inhibited. This could perhaps help to explain the paradoxical similarities between the neurological effects of vitamin B6 shortage and overdose. However, the kinetic rate constants for PN inhibition were not determined in this investigation, and it is still unclear how strongly PN inhibits PLP-dependent enzymes across a range of physiological doses.

In the degradation of tryptophan via the kynurenine pathway, 3-hydroxykynurenine (3-HK) is converted by two PLP-dependent enzymes to xanthate and 3-hydroxy-*o*-toluic acid (3-HAA). 3-HK is a known neurotoxic compound that has been implicated in central nervous system disorders [45, 46]. It implies that one of the main causes of vitamin B6 poisoning that results in neurological problems may be the inhibition of PLP-dependent metabolism of 3-HK by PN, which results in 3-HK accumulation. Replacing PN as a vitamin B6 supplement ingredient with the less toxic PL or PL phosphate might reduce the occurrence of harmful side effects.

The other possibility was put out by Hadtstein and Vrolijk in 2021, who claimed that a plausible hypothesis for the mechanism of toxicity was the inhibition of pyridoxal kinase (PDXK) and the consequent disruption of GABA neurotransmission. Genetic PDXK deficiency can lead to axonal sensory neuropathy, and high doses of PN, which inhibit PDXK function, may create comparable symptoms [36]. Compounds that inhibit PDXK decrease GABA production, which may cause neurological hyperexcitability and seizures, though the precise mechanism by which PDXK causes neuropathy is yet unclear [38, 47]. Because PN is less likely to cross the blood–brain barrier, its inhibitory impact may be confined to PDXK in the periphery, influencing GABA neurotransmission and processing in peripheral tissues, particularly sensory nerves. GABA signaling disruption in sensory neurons may result in excitotoxicity, which can cause neurodegenerative alterations and ultimately promote the development of peripheral neuropathy. However, PDXK inhibition may have a variety of effects, and further experimental research is required to establish the molecular connection between PDXK inhibition and vitamin B6 toxicity.

#### 3.1.4. Clinical Manifestations Associated With Vitamin B6 Overdose

Long-term high-dose intake of vitamin B6 has been associated with sensory neuropathy, with patients presenting mainly with ataxia and sensory nervous system dysfunction [48]. In 1993, the first report of motor neuropathy induced by long-term vitamin B6 misuse was published [49]. The patient's condition improves after discontinuing high doses of vitamin B6, but it may continue to deteriorate for 2 to 3 weeks [50], or the patient may suffer persistent sensory deficiencies [51, 52], which may be related to the high dose.

The neurotoxicity of vitamin B6 has been shown in newborns, the elderly, and even animals. Guala et al. reported a case of spontaneous tremor in a neonate whose mother continued to take high-dose dietary supplements containing vitamin B6 during pregnancy and lactation and whose tremor resolved after stopping the usage of vitamin B6 [53]. The 92-year-old female patient with gait abnormalities was assessed and diagnosed with peripheral neuropathy with motor and sensory deficits, according to a case described by Malet et al. It was caused by the woman self-administering an over-the-counter drug that contained vitamin B6 [54]. In animal experiments, a model of sensory neuropathy created using dogs proved that PN-induced neuropathy was reversible in dogs [55]. It has also been shown that PN exposure may induce seizure-like behavior in animals [56].

In addition, there are some rarer manifestations. Sensory ataxia and loss of lower extremity coordination were observed in a 54-year-old man who had been suffering from gradual, progressive numbness and imbalance for 12 years. That was the first documented case of diffuse sensory ganglionopathy after long-term high PN consumption, according to an electrophysiologic study [57]. Patients with both chronic sensory polyneuropathy and large- and small-fiber neurologic impairments have also been recorded, with uncommon objective evidence of autonomic neuropathy [58]. PDXK mutations can cause axonal sensorimotor polyneuropathy with optic atrophy, and according to Hadtstein's hypothesis of toxicity, PN overdose may cause similar lesions [47, 59]. Some patients have demyelinating symptoms, which might be clinically misdiagnosed as chronic demyelinating polyneuropathy [60].

### 3.2. Vitamin B9

#### 3.2.1. Concept and Intake of Vitamin B9

Folic acid is also a member of the vitamin B family. Folate is the natural form of vitamin B9, while folic acid is the synthetic form. These two forms differ in chemical structure, but both can be converted in the body to the active form, 5-methyltetrahydrofolate (5-MTHF) [61]. Foods containing natural folic acid include yeast, green leafy vegetables, animal liver, and so on. Low folate status is regarded as one of the most prevalent dietary deficiencies owing to a variety of causes, including the scarcity of natural folic acid from food sources, its instability, and sensitivity to bioavailability [62].

Many countries have implemented food fortification to prevent the harmful effects of folate deficiency, especially neural tube defects (NTDs) and megaloblastic anemia [63]. In Ireland, the Netherlands, and Norway, the absolute P95 consumption of folic acid in food supplements ranged from 400 to 600 *μ*g/day. In Germany, it was 893 *μ*g/day for women and up to 1020 *μ*g/day for males. The EFSA established a UL of 1000 *μ*g/day for adults, including pregnant and nursing women. It has been extensively demonstrated that folic acid supplementation effectively prevents prenatal NTDs. Based on solid scientific evidence, the majority of European nations have created public health policies that specifically advise women of childbearing age to take 400 *μ*g of folic acid daily as a supplement from the time of conception until the first trimester of pregnancy. This standard dosage has been confirmed to significantly lower the incidence of NTDs [64]. While folic acid supplementation during the early stages of fetal development is important, particularly neural tube formation, the need to maintain high levels of folic acid supplementation after the first trimester of pregnancy has not been confirmed [65].

Due to the unstable nature of folic acid, dietary intake alone is unlikely to exceed the UL, with the exception of those who frequently take high-dose folic acid supplements. The great stability of synthetic folic acid in supplements, in contrast to natural folic acid, is a significant factor in persons overdosing on vitamin B12 [66]. Long-term overconsumption of folic acid, particularly above 1000 *μ*g/day, may potentially harm the nervous system. When using folic acid supplements for illness prevention and treatment, it is advised that intake be appropriately managed under a doctor's supervision. In order to maximize health benefits and minimize hazards, pregnant women should reevaluate their supplementation needs after mid-pregnancy and refrain from mechanically prolonged high-dose supplementation.

#### 3.2.2. Mechanism of Vitamin B9 Toxicity

The folate cycle is closely linked to the process of one-carbon unit metabolism. If folate levels in the body are excessively high, it can cause an imbalance in the metabolism of one-carbon units, affecting DNA synthesis, repair, methylation, and other important life functions. Here are a few relatively compelling pathways.

Folic acid is converted in the body first to dihydrofolate (DHF) by dihydrofolate reductase (DHFR) and then to tetrahydrofolate (THF) [62]. In the human body, the enzymatic activation of folate by DHFR is slow, and when FA intake reaches a certain limit, the rate-limiting enzyme DHFR becomes saturated, resulting in the accumulation of unmetabolized folic acid (UMFA) in the circulation [67]. Several studies have shown that increased FA intake causes higher levels of circulating UMFA [68, 69]. In order to participate in one-carbon metabolism, THF must be converted by SHMT, which requires PLP as a cofactor, to 5,10-methylenetetrahydrofolate, which is subsequently catalyzed by MTHFR to 5-MTHF. Competitive inhibition of DHFR activity by UMFA leads to the accumulation of DHF, which is a potent inhibitor of MTHFR [66], ultimately disrupting the balance of the one-carbon cycle. Some of the reactions in the cycle may be dysregulated, affecting DNA synthesis and repair, as well as methylation processes.

Methionine synthase (MTR) catalyzes the conversion of homocysteine to methionine and the synthesis of THF using 5-MTHF as a substrate. A vitamin B12 deficiency inhibits MTR function and causes 5-MTHF to accumulate since this process depends on vitamin B12 as a coenzyme. When vitamin B12 deficiency and excess folate combine, a special metabolic state known as a methyl trap is created [66]. A functional folate shortage is caused when the majority of the body's folate is found in 5-MTHF since intracellular THF is reduced. Decreased THF hinders the synthesis of purines and pyrimidines, which in turn impacts the synthesis of DNA and RNA [62]. Megaloblastic anemia, which is characterized by large, immature red blood cells, is the result of a disturbance of nucleotide generation. Excessive folic acid supplementation can mask the hematologic symptoms of vitamin B12 deficiency, thereby delaying diagnosis and leading to more severe or even irreversible neurologic symptoms. Furthermore, vitamin B12 deficiency and impaired MTR activity lead to homocysteine accumulation, and elevated levels may increase the risk of cardiovascular disease, neurological illness, and other negative health outcomes [70–72].

As mentioned earlier, high folate levels inhibit MTHFR, which affects subsequent methionine synthesis and significantly lowers the methyl donor SAM. SAM acts as both a cofactor and a methyl donor in a range of processes, including the methylation of DNA, RNA, neurotransmitters, and small molecules. These SAM-dependent reactions play important regulatory roles for genome stability, gene transcription, protein localization, and small molecule degradation [73]. High folate levels can disrupt the metabolism of folate, which can result in DNA hypomethylation, epigenetic instability, abnormal gene expression, and eventually the development and spread of cancer [66].

Although a connection between excessive maternal FA intake and offspring defects has been shown, there is no direct proof that high folic acid intake leads to aberrant DNA methylation and changes in gene expression. DNA methylation is a complicated and highly controlled biological process, and current research does not support a simple linear or dose–response relationship between folic acid consumption and specific DNA methylation levels. It is uncertain if other unknown routes or the direct disruption of one-carbon metabolism caused by high FA is responsible for the ensuing epigenetic alterations. Therefore, more research is required to elucidate the connection between folate and DNA methylation.

#### 3.2.3. Clinical Manifestations Associated With Vitamin B9 Overdose

Excessive folate intake may have neurodevelopmental effects in the offspring, has been strongly associated with an increased risk of autism spectrum disorders (ASDs) and epilepsy, and affects cognitive function in combination with vitamin B12.

Excessive folic acid intake exhibits neurodevelopmental toxicity. It disrupts methylation and alters brain gene expression and behavioral patterns in mouse offspring, with some genes showing significant sex specificity [74, 75]. By influencing epigenetic modifications, excessive folic acid supplementation may interfere with children's cognitive development [76]. To determine the appropriate folic acid dose, the upper limit of folic acid consumption, and the best time to use folic acid supplements for neurodevelopment, more research is required [77].

Excessive folate intake may be associated with an increased risk of ASD. According to certain research, high maternal plasma folate levels may considerably raise a child's risk of developing ASD [78, 79]. On the other hand, a modest intake of 400 *μ*g/day of folic acid from diet and supplements during pregnancy may lower the probability that the baby would have ASD [80]. This implies that there is a “U-shaped” association between the risk of ASD and maternal folic acid supplementation; either excessive or insufficient folic acid consumption may raise the risk of ASD in children [81]. In fact, the relationship between folic acid supplementation and ASD is complicated; some research has found no connection at all [82]. In order to better analyze the potential role of folic acid in the risk of ASD, more rigorous clinical studies with bigger sample numbers and longer follow-up periods are required.

There is evidence that folic acid lowers the levels of antiepileptic drugs (ASMs). Folic acid is a cofactor for phenytoin sodium metabolism and may produce seizures in patients who take phenytoin sodium. One case vividly indicates that the relationship between folic acid and phenytoin sodium metabolism is dual and interdependent [83]. Besides, increased folic acid intake may diminish the concentration of carbamazepine and phenobarbital in the blood, thus increasing the risk of seizures [61, 84]. The ideal dosage of folic acid supplementation for women with epilepsy of reproductive age who are on antiepileptic drugs is up for debate; recommended doses range from 0.4 to 5 mg [85]. It is advised that the specific amount of folic acid supplements should be adjusted to the maternal plasma folic acid level throughout pregnancy. A study found that high folic acid intake during pregnancy may accelerate neuronal development and create hyperexcitable networks in the nervous system, increasing the offspring's risk of seizures [65].

To avoid masking the signs of a B12 deficiency and preventing possible neurologic damage, it is crucial to take vitamin B12 status into consideration and be aware of methyl traps when taking folic acid supplements. Vitamin B12 deficiency-induced megaloblastic anemia can be partially resolved by folic acid supplementation, which may delay the detection and treatment of the condition [86]. However, it does not prevent the neuropathy associated with cobalamin deficiency and its harmful neurologic consequences caused by high methylmalonic acid levels [61]. Furthermore, high serum folate concentrations are related to an increased risk of cognitive impairment in patients with hypovitaminosis B12, but normal vitamin B12 status is associated with a lower risk of cognitive impairment in the presence of high serum folate [87].

## 4. Conclusions

Despite the fact that vitamins have been discovered and studied for more than a century, our understanding of their bioactivity requires further refinement. The results of several experimental studies and clinical trials are conflicting and contradictory, and it is possible that the bioactivity of vitamins is influenced by some unknown factors, such as age, gender, and epigenetic inheritance. Future research should continue to explore the specific mechanisms of neurotoxicity associated with vitamin overdose intake, identify genetic factors that may influence individual susceptibility, and design more effective interventions. Maintaining appropriate levels of vitamin intake is critical to avoiding correlated health problems, and it is especially necessary to establish effective preventive methods. Patients should take additional vitamins under a doctor's supervision and have their neurological condition regularly checked. Clinicians should be aware of the possible toxicity of vitamins, focus on the use of supplements for diagnosis and treatment, and warn patients about the risks of using large amounts of these products.

## Data Availability

The authors have nothing to report.
